# Straw vs Cup Use in Patients with Symptoms of Oropharyngeal Dysphagia

**DOI:** 10.51894/001c.11591

**Published:** 2020-01-30

**Authors:** Bo Pang, Paul Cox, Julianna Codino, Austin Collum, Jake Sims, Adam Rubin

**Affiliations:** 1 Otolaryngology Ascension Macomb-Oakland Hospital; 2 Ear, Nose, and Throat PC Lakeshore Professional Voice Center; 3 Otolaryngology, Ear, Nose, and Throat PC Ascension Macomb-Oakland Hospital, Lakeshore Professional Voice Center

**Keywords:** oropharyngeal, swallow, straw, cup, fees, dysphagia

## Abstract

**CONTEXT:**

This study aims to determine whether straw or cup use is superior for the control of a single thin liquid bolus in patients with symptoms of oropharyngeal dysphagia to liquids.

**METHODS:**

This is a prospective, randomized, single-blinded study. Patients were studied at a Professional Voice and Swallowing Center by a laryngologist between April 2017 and April 2018. Twenty-five patients, 18 years of age or older, who presented with symptoms of oropharyngeal dysphagia the clinic were included in the study. Each patient complained of difficulty with choking on liquids. Informed consent was obtained from each patient. Patients that were unable to follow one to two step commands and patients with dysphagia that lack oral strength or respiratory strength to facilitate straw or cup usage were not included. Patients with dysphagia that are tracheostomy tube dependent were also not included.

**RESULTS:**

The average PAS for straw versus cup drinking at 10mL was 1.08 and 1.04 respectively with a p-value of 0.33. For straw versus cup at 20mL, the PAS was 1.04 and 1.26 respectively with a p-value of 0.13. For 30mL, the PAS was 1.0 and 1.4 for straw and cup use respectively with a p-value of 0.16. And for 40mL, the PAS was 1.0 and 1.09 with a p-value of 0.27.

**CONCLUSIONS:**

No statistical significant difference was demonstrated in risk of penetration or aspiration of thin liquids between cup and straw usage in patients with mild oropharyngeal dysphagia.

## Introduction:

Dysphagia, derived from the Greek words “dys” and “phagia”, is the medical term that describes difficulty swallowing. Dysphagia affects around 2-11% of the general North American population.^1^ There are multiple causes of dysphagia, the most common of which differ by age groups.^2^ In the middle-aged population, the most common culprits of dysphagia are immunological and gastroesophageal causes, whereas in the elderly population, dysphagia is most commonly caused by oncologic or neurologic factors. These factors include sarcopenia, cerebrovascular accidents, Parkinson's disease, motor neuron diseases, and iatrogenic causes as a result of intubation or effects of head and neck cancer treatments.

Dysphagia may involve one or several of the phases of swallowing. Determining the most likely phase of swallowing involved is necessary to determine the appropriate workup. This is best achieved through detailed history-taking. Oropharyngeal dysphagia is best evaluated with a modified barium swallow (MBS) or flexible endoscopic evaluation of swallowing (FEES). Symptoms of oropharyngeal dysphagia might include coughing during or after the swallow or early obstructive symptoms. Sometimes aspiration can be silent, and only recognized with MBS or FEES. More severe cases may present with recurrent pneumonia or weight loss.

Straw use in the patient with oropharyngeal dysphagia is controversial. Patients are often instructed to use a cup for oral liquid intake, however, the literature surrounding the use of cup over straw for dysphagic patients has been limited. Most studies conducted on the physiology of swallowing and straw use have been on healthy or elderly adults who do not complain of swallowing difficulties. Many patients with liquid bolus control issues and premature spillage are instructed to tuck the chin.^3^ Ingestion of liquid with a chin tuck is difficult without a straw.

Veiga, *et al.* have demonstrated that straw use in the elderly has a more favorable influence on the oral phase of the sequential swallowing of liquid when compared to cup use.^4^ Contradictory to that, Daniels *et al.* showed that sequential straw drinking allows the bolus to drop too low in the pharynx before the onset of the swallow - resulting in greater risk of airway compromise.^5^ The average single bolus size for thin liquids is reported to be around 20-25mL in adults.^6^ There is an increase in risk of aspiration with increase in bolus volume.^7^ This study aims to determine whether straw or cup use is superior for the control of a single thin liquid bolus in patients with symptoms of oropharyngeal dysphagia to liquids.

## Methods:

This is a prospective, single-blinded study. The study was approved by the St. John-Providence IRB Board. 25 patients, 18 years of age or older, who presented with symptoms of oropharyngeal dysphagia to Lakeshore Ear, Nose and Throat Center January 1^st^ to February 28^th^, 2018 were included in the study. Each patient complained of difficulty swallowing thin liquids, and frequent choking. Informed consent was obtained from each patient. Patients that were unable to follow one to two step commands and patients with dysphagia that lacked oral strength or respiratory strength to facilitate straw or cup usage were not included. Patients with dysphagia that were tracheostomy tube dependent were also not included. Patient demographics were recorded, but all key identifiers removed.

A complete history and head and neck examination were performed on every patient by a fellowship-trained laryngologist. Patients then underwent FEES. Patients were asked initially to take a normal sip of water from two conical cups containing 50mL of water each; once with a straw and once without. The remaining liquid volume was used to calculate the average bolus volume via straw and cup, respectively.

FEES was then performed in the standard fashion.^8^ However, initially, each patient was given a thin liquid trial with 4 different volumes (10mL, 20mL, 30mL, and 40mL), with and without straw use. They were instructed to consume in one bolus. Their swallows were recorded on the Kay-Pentax video recording system. Patients that could not tolerate the smaller volumes were not given any greater volume of liquid.

Video recordings for each patient were cropped and labeled with patient number and letter “a” to “h”. Letters were randomly assigned to different liquid trials and each video was stripped of sound or any identifying information. The videos were given to three speech-language pathologists blinded to the purpose of the study and method of ingestion. Using the Penetration and Aspiration Scale (PAS), each speech-language pathologist individually rated each patient’s swallow and assigned a numeric value of 1 to 8. This scale was used based on its great inter- and intra-rater reliability.^9^ Although the PAS was originally created for videofluoroscopy, the scale is equally as reliable when being used for FEES.^10^ All three reviewers scores were then analyzed individually as well as combined for the main outcome variables indicated below.

Differences in PAS scores between straw and cup ingestion, volume of bolus, and average bolus size were evaluated. Statistical analysis was performed using Chi-Square (Fisher’s Exact) test for discrete variables and the Student’s T-test for continuous variables. SPSS testing was used. A p-value of less than 0.05 was considered significant.

## Results:

A total of 25 patients were included in this study. The average age of the patient was 66 [range 41-92]. 15/25 (60%) were female, and 10/25 (40%) were male. The average bolus size for these patients was 16.7mL for cup use and 14.1mL for straw use. One patient could not finish 30mL of liquid for either straw or cup drinking so 40mL was not measured. Two patients required the chin-tuck maneuver to prevent premature spillage during swallows.

The average PAS for straw versus cup drinking at 10mL was 1.08 (SD 0.28) and 1.04 (SD 0.20) respectively (p = 0.327). For straw versus cup at 20mL, the average PAS was 1.04 (SD 0.03) and 1.26 (SD 0.62) respectively (p = 0.133). For 30mL, the average PAS was 1.13 (SD 0.00) and 1.40 (SD 1.38) for straw and cup use respectively (p = 0.162). For 40mL, the average PAS was 1.06 and 1.09 respectively (p = 0.264).

**Graph 1:**
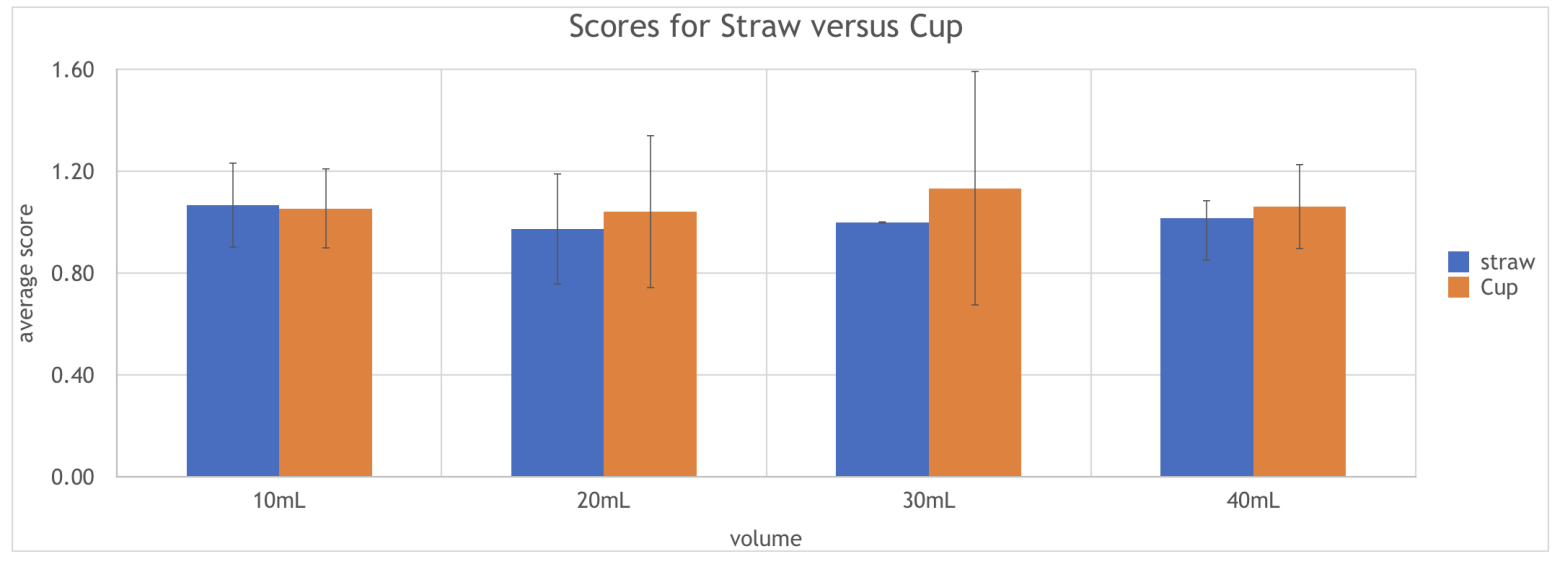
Average PAS scores for straw vs cup at different volumes of liquid

These findings indicate that although there is a slight increase in the PAS scoring for cup use at 20mL, 30mL, and 40mL, the difference was not statistically significant. As well, all scores were close or equal to 1 for either utensils for all four volumes of liquid, indicating that contrast does not enter the airway and that there was no aspiration for the majority of the swallows in our patient cohort. *See* graph 1.

For straw use, there was no change in the PAS scoring between the four different volumes of swallow. For cup use, as cup volume increases, there is a slight increase in the PAS scoring; However, although it appears that it is more difficult to drink out of a cup at larger volumes, the difference was not statistically significant.

One patient in our study did not finish the 30mL swallow for cup or straw drinking due to physical discomfort, therefore the 40mL swallows were not performed. This patient’s 10mL and 20mL data were still included in this study. Notably, this patient still had scores of 1 on the PAS for both methods during these trials.

## Discussion:

There was no significant difference in the PAS scores between cup and straw usage at 10mL, 20mL, 30mL, or 40mL volumes in this cohort of patients. This is the first study to compare aspiration risk between straw and cup use in patients complaining of difficulty swallowing liquids. Studies have shown that in healthy elderly patients, cup drinking allows for a larger total volume of intake per bolus, whereas straw drinking allows for better bolus containment within the oral cavity with less oropharyngeal spillage.^4^ This same study also shows that the PAS scores for healthy elderly patients for both cup and straw use results in a PAS of 1 in 96% of patients. This study further enforces the current literature to include patients with symptoms of oropharyngeal dysphagia to liquids, and suggests that the aspiration risk in a mildly dysphagic patient, regardless of the bolus delivery method, is low.

This indicates that even for patients who experience dysphagia at higher volumes of liquid, aspiration remains a low threat for both cup use and straw use for patients.

There are several limitations to this study. The study looked at patients who complained of difficulty with swallowing liquids. However, as can be seen from the PAS scores, these patients did not have documented aspiration. Future studies looking at subsets of patients at greater risk for aspiration of liquids (stroke patients, for example) would prove useful. One could argue that the PAS may be better utilized for more severe patients that have documented penetration prior to the study. In addition, many more severely dysphagic patients require thickening of fluids to prevent aspiration. Straw use in this population presents unique challenges to control for dependent upon, etiology of impairment, impairment severity and the level of thickness required to decrease the risk of airway compromise (i.e. nectar thick liquids versus honey thick liquids). In addition, this study only looked at PAS scores which are limited to penetration and aspiration only. This study does not include symptoms such as the cough reflex, choking symptoms, or premature spillage. For example, straw use would be the more preferred utility in performing chin-tuck maneuvers in patients with premature spillage.

Another potential limitation of this study is the so-called “whiteout effect” during FEES. The whiteout effect occurs during the oropharyngeal phase of swallowing, where the bolus, the endoscope, epiglottis, and the base of tongue are in contact and limits the view from the endoscope during the initiation of the swallow reflex.^11^ The whiteout effect may limit and underestimate the degree of penetration or aspiration during evaluation by the blind readers. However, when the whiteout effect is strong, it usually is due to a timely swallow. It is less likely to be seen in higher-risk patients with premature spillage of liquids, for example.

## Conclusion:

No statistical significant difference was demonstrated in risk of penetration or aspiration of thin liquids between cup and straw usage in patients with mild oropharyngeal dysphagia. Future studies comparing differences in straw and cup ingestion in patients with more severe dysphagia are necessary. As well, studies with a higher patient population would be necessary for an increase power.

### Conflict of Interest

The authors declare no conflict of interest.
